# Linking Transformational Leadership to Patient Care Quality: The Role of Structural Empowerment and Registered Nurses’ Clinical Leadership

**DOI:** 10.1155/jonm/7518764

**Published:** 2026-05-20

**Authors:** Abdulaziz K. Alanazi, Marian Traynor, Lisa McFetridge, Clare McKeaveney, Susan A. Clarke

**Affiliations:** ^1^ School of Nursing and Midwifery, Queen’s University Belfast, Belfast, UK, qub.ac.uk; ^2^ Nursing Administration, King Saud Medical City, Riyadh, Saudi Arabia, ksmc.med.sa; ^3^ Mathematical Sciences Research Centre, School of Mathematics and Physics, Queen’s University Belfast, Belfast, UK, qub.ac.uk

## Abstract

**Background:**

Effective leadership is critical in driving and motivating registered nurses to provide high‐quality patient care. A link between effective transformational leadership (TL) in nursing and improved patient care outcomes has been established, but it is unknown how this association is affected.

**Aim:**

To examine the relationships between TL, structural empowerment (SE), registered nurses’ clinical leadership (CLS) and patient care outcomes.

**Methods:**

This quantitative cross‐sectional study was conducted in a government tertiary hospital in Saudi Arabia, with 1038 registered nurses clustered under 28 head nurses. Instruments used included the Multifactor Leadership Questionnaire, Conditions of Work Effectiveness Questionnaire II, clinical leadership survey and self‐reported measures of patient adverse events (AEs) and nursing care quality. Data were analysed using SPSS Version 28 and the R 4.3.3 packages ‘lavaan’, ‘semPlot’ and ‘semTools’ for multilevel structural equation modelling (MSEM).

**Results:**

Six hundred and sixty‐four surveys (response rate = 70%) were analysed. Multilevel analysis showed a significant positive association between TL and SE (*β* = 0.56, *p* < 0.001) and SE and registered nurses’ clinical leadership (*β* = 0.52, *p* < 0.001). Registered nurses’ clinical leadership was positively associated with quality of care (*β* = 0.32, *p* < 0.001) and negatively associated with AEs (*β* = −0.15, *p* < 0.001). Mediation analysis indicated a significant indirect effect of TL on registered nurses’ clinical leadership through SE (*B* = 0.344, *p* < 0.001). SE was also indirectly associated with patient AEs through registered nurses’ clinical leadership (*B* = −0.037, *p* = 0.003) and quality of nursing care (QOC) through registered nurses’ clinical leadership (*B* = 0.147, *p* < 0.001).

**Conclusions:**

The study highlights the critical role that SE and nurses’ clinical leadership play in explaining how TL is associated with improved patient care. These findings provide evidence to support policy and training initiatives that develop leadership frameworks, ensuring a more empowered and effective nursing workforce capable of delivering high‐quality patient care.

## 1. Introduction

In the complex and rapidly evolving landscape of healthcare, effective leadership is an essential aspect in ensuring high‐quality patient care and optimal organisational performance. Transformational leadership (TL), defined as a style whereby leaders inspire and motivate employees to exceed expectations by transforming their attitudes and beliefs [1], enhances organisational commitment, empowerment, job satisfaction, motivation, trust and self‐efficacy among staff members [2–6]. This leadership style encompasses four key components: idealised influence, inspirational motivation, intellectual stimulation and individualised consideration.

In healthcare settings, TL not only improves patient care quality but also increases staff retention by reducing turnover rates [7–11]. Given the critical role that nurses play as direct care providers, evaluating and supporting TL within nursing units is essential to ensure high‐quality care and patient safety [7, 9, 12].

Structural empowerment (SE), grounded in Kanter’s theory [13], refers to organisational structures that provide employees with access to information, resources, support and the opportunities necessary to perform their roles effectively. Empowered nurses experience greater autonomy and support within their work environment, leading to increased job satisfaction and reduced adverse patient events [3, 12, 14, 15]. Transformational leaders facilitate SE by creating supportive environments that enable nurses to excel in their roles.

Clinical leadership involves the provision of leadership in clinical settings to ensure that patient care is delivered safely, efficiently and effectively [16]. Nurses who exhibit clinical leadership take active roles in coordinating patient care plans, setting goals and collaborating within teams, directly affecting patient care quality. Such leadership at the bedside is crucial, yet current research often overlooks the impact of individual nurses in informal leadership roles on care quality and outcomes [17].

Despite the well‐documented benefits of TL, the mechanisms through which it influences patient care outcomes remain unclear. Specifically, the mediating roles of SE and registered nurses’ clinical leadership have not been sufficiently explored. Previous research has examined the relationships between TL, SE and clinical leadership using individual‐level self‐report survey data. However, such data are inherently limited, capturing only individual exposures and responses, which restricts the ability to account for broader contextual influences [18–20]. To address this limitation, the current study employs a multilevel analytical approach, enabling a more comprehensive exploration of the interplay between TL, SE and clinical leadership across diverse healthcare settings.

Furthermore, demographic factors such as gender and nationality may influence leadership behaviours and outcomes in healthcare settings. Female leaders, for instance, tend to adopt TL styles more frequently, which are associated with better job satisfaction and patient outcomes [21]. Cultural backgrounds can affect the efficacy of leadership practices, highlighting the importance of considering these variables when studying leadership outcomes [22, 23]. Understanding how TL interacts with SE and clinical leadership within diverse healthcare environments is critical to addressing pressing challenges in nursing leadership. By examining these relationships, this research aims to guide strategies that foster effective leadership, improve patient care outcomes and advance healthcare systems worldwide.

## 2. Objectives

The aim of this study was to examine the relationships between TL, SE, registered nurses’ clinical leadership and patient care outcomes as perceived by registered nurses. By testing hypotheses derived from Bass’s TL theory, Kanter’s SE theory and Patrick’s clinical leadership construct, this research sought to fill the gap in understanding the mediating roles of SE and clinical leadership. Additionally, the study examined how demographic factors, such as gender and nationality, affect these relationships within healthcare settings.

### 2.1. The Hypothesised Theoretical Model

1. H1: TL of head nurses will be positively associated with SE.
2. H2: SE will be positively associated with the clinical leadership of registered nurses (CLS).
3. H3: Higher clinical leadership by registered nurses (CLS) will be positively associated with quality of patient care (QOC).
4. H4: Higher clinical leadership by registered nurses (CLS) will be negatively associated with AEs.
5. H5: SE mediates the association between TL and the clinical leadership of registered nurses (CLS).
6. H6: Clinical leadership by registered nurses (CLS) mediates the association between SE and QOC.
7. H7: Clinical leadership by registered nurses (CLS) mediates the association between SE and patient AEs.
8. H8: SE and the clinical leadership (CLS) of registered nurses together mediate the association between TL and patient AEs.
9. H9: SE and clinical leadership (CLS) jointly mediate the association between TL and the QOC.

## 3. Methodology

### 3.1. Study Design and Participants

This quantitative study employed a cross‐sectional design and was reported in accordance with the Strengthening the Reporting of Observational Studies in Epidemiology (STROBE) Statement for cross‐sectional studies. It was conducted in a government tertiary hospital in Riyadh, Saudi Arabia. All registered nurses who were working under a random selection of head nurses were invited to participate, provided they met the inclusion criteria of working at the bedside, providing direct patient care and having been employed for at least 3 months. A stratified cluster sampling method was used, in which clusters were stratified by the nationality and gender of the head nurses to ensure diversity and representativeness of the sample. This stratification allowed for an examination of how these demographic variables may influence the adoption of TL and its impact on clinical outcomes. A total of 1038 nurses were invited to participate from 28 units, led by 28 head nurses, to capture a comprehensive range of perspectives across different demographic groups within the nursing staff. Measures such as ensuring the anonymity of responses and employing stratified cluster sampling were taken to minimise potential sources of bias and enhance the study’s validity and reliability.

### 3.2. Data Collection

Data were collected between 26 December 2021 and 31 January 2022, at a government tertiary hospital in Riyadh, Saudi Arabia. Registered nurses were invited to participate in the study through a combination of emails, flyers and WhatsApp messages containing a QR code or survey link to access the online questionnaire hosted on Qualtrics. To enhance participation and account for nurses who were on leave during the initial recruitment period, follow‐up messages were sent 2 weeks after the initial invitation. After 3 weeks, the data collection period was extended by an additional 2 weeks, during which a final reminder was sent 1 week before the survey closed. This approach ensured that all eligible nurses had ample opportunity to participate in the study.

### 3.3. Sample Size Calculation

The sample size was calculated based on Bentler and Chou’s guideline [24], which recommends a ratio of 10 participants per parameter. This resulted in a required sample size of 560 participants for the model. To account for an anticipated 50% response rate, 1038 nurses were invited to participate and, ultimately, 664 valid surveys were returned. This exceeded the minimum requirement, ensuring sufficient data to power the analysis [25, 26].

### 3.4. Measurements

#### 3.4.1. Multifactor Leadership Questionnaire (MLQ)

To assess the TL qualities of head nurses, this study utilised 20 questions using the MLQ. The MLQ is a widely recognised and extensively researched instrument that has proven valid and reliable for studying diverse settings, including Saudi Arabia [22, 27, 28]. There were five dimensions to the TL questionnaire, including idealised influence attributes, idealised influence behaviours, inspirational motivation, intellectual stimulation and individual consideration, and each had four items. Each item was rated on a Likert scale from 0 (not at all) to 4 (frequently, if not always), which were summed and averaged. The reported Cronbach’s alpha for the TL scale was 0.97 [14], the same as that recorded in the current study.

#### 3.4.2. Conditions of Work Effectiveness Questionnaire II (CWEQ‐II)

SE was evaluated through the CWEQ‐II, which consists of 12 items [1, 29]. The CWEQ‐II measured nurses’ access to work empowerment structures, including opportunity, information, support and resources, as described in Kanter’s structural theory of organisational empowerment (1977) [13]. Each subscale contained three items rated on a five‐point scale, where 1 denotes Strongly Disagree, 2 denotes Disagree, 3 denotes Neutral, 4 denotes Agree and 5 denotes Strongly Agree, and these were summed and averaged. Reported Cronbach’s alphas for this scale ranged from 0.74 to 0.89 [29]. In this study, the Cronbach’s alpha was calculated at 0.92.

#### 3.4.3. Clinical Leadership Survey (CLS)

The clinical leadership survey, as established by Patrick et al. [16], was utilised to assess the clinical leadership of registered nurses. Comprising 15 items distributed across five subscales—challenge the process (CPP), inspiring a shared vision (ISV), enabling others to act (ICT), modelling the way (MOW) and encouraging the heart (HER)—this tool is designed to gauge perceptions of clinical leadership at the staff nurse level [12]. Each item was rated on a Likert scale of 1 (almost never) to 5 (almost always), which were summed and averaged. The reported Cronbach’s alpha reliability coefficient for the total registered nurses’ clinical leadership scale is 0.86 [16], but in this study, it was calculated as 0.93.

#### 3.4.4. QOC

The QOC was evaluated using two instruments. The first, developed by Aiken et al. [30], measured the self‐reported frequency of patient AEs. Registered nurses rated the occurrence of specific AEs (medication errors, patient falls with injuries, healthcare‐associated infections and complaints from patients or their families) over the past year on a 4‐point Likert scale from never [1] to frequently [4]. Higher scores indicated a greater frequency of these events.

The second instrument, the QOC questionnaire, comprised four questions assessing nurses’ perceptions of care quality on their unit. This covered their last shift, overall changes in care quality over the past year and their confidence in patients’ ability to manage their care postdischarge [31, 32]. Responses to the first two questions and the last question were rated on a four‐point Likert scale from Poor [1] to Excellent [4] and from not at all confident [1] to very confident [4], respectively. The third question asked participants to evaluate whether the QOC had deteriorated, remained the same or improved. In this study, the reliability of these instruments was supported by Cronbach’s alphas of 0.83 for the AEs questionnaire and 0.77 for the QOC questionnaire.

### 3.5. Ethical Approval

This study obtained ethical approval from Queen’s University Belfast (QUB), the MHLS Faculty Ethics Committee and the Institutional Review Board (IRB) at KSMC in Riyadh (the Ethical Approval Number is QUB: MHLS 21_80 and KSMC: H1RI‐11‐Aug21‐0). Participation was voluntary, and informed consent was implied through the completion of the survey after participants had read the study information sheet.

### 3.6. Statistical Methods

Data were analysed using IBM SPSS Statistics Version 28 for descriptive analysis [33]. Following this initial analysis, the R (Version 4.3.3) [34] packages ‘lavaan’ [35], ‘semPlot’ [36] and ‘semTools’ [37] were employed to conduct multilevel confirmatory factor analysis (CFA) and multilevel structural equation modelling (MSEM). All survey questions were mandatory, resulting in no missing data. The study used maximum likelihood estimation in the R packages for the SEM. Given the hierarchical structure of the data, with individual nurses (lower level units) nested within hospital units (higher level units), multilevel modelling was employed to account for potential similarities among nurses within the same unit [38, 39]. This approach is essential in healthcare settings to accurately examine outcomes such as TL, SE, clinical leadership, adverse patient events and QOC.

In SPSS, descriptive statistics summarised all variables, while exploratory multilevel analyses examined the influence of demographic factors on key variables across different levels in the data. This exploratory phase allowed for a comprehensive understanding of demographic factors at the unit level, which interact within a nested data structure. These exploratory analyses provided initial insights for interpreting relationships prior to formal hypothesis testing [40–42].

For the formal hypothesis testing phase, MSEM in R examined the relationships between latent variables across different levels of data. Gender and head nurse nationality were retained as control variables due to their significant impact on leadership dynamics and healthcare outcomes, as documented in prior research [21, 22]. Other potential confounders, such as unit size (number of beds), years of experience of head nurses and staff shortages, were analysed descriptively during the exploratory phase, but were excluded from the final models to avoid overcomplicating the analysis and to maintain focus on the core relationships under investigation.

The decision to retain only gender and nationality aligns with the principle that covariates should be selected based on their known influence on either the exposure or the outcome [43, 44]. While other factors, such as unit size and staff shortages, were important for contextual understanding, their inclusion risked overfitting the model and introducing unnecessary complexity. This approach also ensured comparability with other studies that have highlighted gender and nationality as key determinants of leadership practices and staff perceptions in healthcare settings. In line with the study’s conceptual pathway, unit workload, staffing ratios and patient acuity were not included as covariates in the structural models. These factors lie close to the outcomes and form part of the same mechanism linking leadership, SE and clinical leadership to patient results. Controlling for them can obscure the indirect relationships that the mediation analysis aims to examine [40, 41]. Consistent with prior evidence linking higher patient acuity and staffing pressures to increased burnout, task demands and reduced quality of care [45, 46], these variables were treated as contextual descriptors rather than exogenous covariates to maintain a parsimonious and interpretable model.

This study assessed the measurement model to establish the validity and reliability of the constructs. The structural model was used to test a model linking TL and SE to registered nurses’ clinical leadership and patient care outcomes. *p* Values < 0.05 were considered statistically significant using a two‐tailed test.

The model fit indices were examined to assess the overall goodness‐of‐fit. Various fit indices were used to assess model‐data fit. The comparative fit index (CFI) is a commonly used fit statistic, with values above 0.90 indicating an acceptable fit and 0.95 or higher suggesting a good fit [47]. The root‐mean‐square error of approximation (RMSEA) measures the extent of lack of fit, with values of 0.01, 0.05 and 0.08 indicating an excellent, good and adequate fit, respectively [47]. The standardised root‐mean‐square residual (SRMR) is another important fit index, where values of 0.08 or lower are desirable. However, this index is sensitive to model misspecifications, with larger values indicating a worse fit [47]. For the Tucker–Lewis index (TLI), values above 90 indicate an acceptable fit and values of 95 or higher are indicative of a good fit. These indices provided insights into the degree of similarity between the hypothesised model and the observed data. Finally, to assess the risk of common method bias (CMB) due to the use of self‐report data from a single survey, Harman’s single‐factor test was conducted. The first factor accounted for 31.7% of the variance, below the 50% threshold, suggesting that CMB was not a major concern [48, 49].

## 4. Results

Six hundred and sixty‐four surveys (response rate = 70%) were analysed after excluding 71 participants due to entries from nontarget units, very brief completion times and straight‐lining patterns, where participants selected the same answer across all questions. Most registered nurse participants were female (95%), and more than half were 26–35 years old (see Table 1). The correspondents’ level of nurse education was also captured at four levels—Diploma in Nursing, Bachelor of Nursing, Master of Nursing and PhD—and the majority of participants were educated to Bachelor’s level. Participants had been randomly selected across speciality units including the Emergency Department (22.9%), Critical Care (36.3%), General Ward (31.5%), Catheterisation Laboratory (7%), Operating Rooms (1.4%) and Haemodialysis (6.3%).

**TABLE 1:** Demographic characteristics of participants.

Subcategory	Number of responses (response rate)	Sample percentage (%)
Gender		
Female	632 (65.0%)	95.0
Male	32 (48.0%)	5.0
Ethnicity		
Saudi	116 (41.0%)	17.5
Filipino	254 (70.0%)	38.3
Indian	282 (76.0%)	42.5
Other	12 (46.0%)	1.8
Age		
20–25	22	3.3
26–30	149	22.4
30–35	255	38.4
36–40	130	19.6
40–45	63	9.5
46–50	29	4.4
> 50	16	2.4
Education		
Diploma	125	18.8
Bachelor’s	522	78.6
Master’s	17	2.6
PhD	0	0
Unit Specialty		
Emergency Department (ER)	152 (64.4%)	22.9
Critical Care (ICU)	241 (85.1%)	36.3
General Ward (GW)	209 (57.7%)	31.5
Catheterisation Laboratory (Cath lab)	11 (36.6%)	1.7
Operating Rooms (OR)	9 (17.6%)	1.4
Haemodialysis	42 (55.2%)	6.3

Table 2 presents the demographic characteristics and experience levels of the 28 head nurses overseeing the units included in the study. Among the head nurses, 67.9% were female (*n* = 19) and 32.1% were male (*n* = 9). Females had a higher average experience as registered nurses (8.58 years, SD = 4.66) compared to males (4.71 years, SD = 1.92). Conversely, male head nurses had more experience in their head nurse roles (9.58 years, SD = 5.70) than females (4.91 years, SD = 2.59). The total years of nursing experience were similar between females (14.30 years, SD = 4.54) and males (13.49 years, SD = 5.32). Regarding nationality, Saudis comprised the largest group of head nurses at 42.8% (*n* = 12), followed by Filipinos at 32.1% (*n* = 9), Indians at 14.2% (*n* = 4) and Jordanians at 10.7% (*n* = 3). Indian head nurses had the highest average years of experience as registered nurses (12.70 years, SD = 5.30) and the highest total years of experience (18.05 years, SD = 3.96). Jordanians had the most experience as head nurses (13.83 years, SD = 4.50). In terms of Unit Specialty, over half of the head nurses (52%) were assigned to the General Ward (*n* = 15). The Emergency Unit and Critical Care Unit each had 17% of the head nurses (*n* = 5 for each). The Catheterisation Lab and Operating Rooms each had one head nurse (3%), and the Haemodialysis units had two head nurses (7%).

**TABLE 2:** Demographics of the head nurses of the units sampled in the study retrieved from official hospital records (*n* = 28).

Subcategory	*n* (%)	Years of experience as a registered nurse mean (SD)	Years of experience as a head nurse mean (SD)	Total years’ experience mean (SD)
Overall mean (SD)		6.88 (4.17)	6.96 (4.83)	13.85 (5.01)
Gender				
Female	19 (67.9%)	8.58 (4.66)	4.91 (2.59)	14.30 (4.54)
Male	9 (32.1%)	4.71 (1.92)	9.58 (5.70)	13.49 (5.32)
Nationality				
Saudi	12 (42.8%)	6.60 (3.59)	4.90 (2.13)	11.50 (4.97)
Filipino	9 (32.1%)	8.72 (3.43)	5 (3.19)	13.73 (3.60)
Indian	4 (14.2%)	12.70 (5.30)	5.34 (3.50)	18.05 (3.96)
Jordanian	3 (10.70%)	3.85 (2.00)	13.83 (4.50)	17.68 (2.50)
Unit Speciality of Head Nurses				
Emergency Unit (ER)	5 (17%)	—	—	—
Critical Care (ICU)	5 (17%)	—	—	—
General Ward (GW)	15 (52%)	—	—	—
Catheterisation Laboratory (Cath Lab)	1 (3%)	—	—	—
Operating Rooms (OR)	1 (3%)	—	—	—
Haemodialysis	2 (7%)	—	—	—

Table 3 presents the key findings on TL, SE, clinical leadership, patient AEs and quality of care. TL scored moderately (*M* = 3.01, SD = 0.82), with head nurses excelling in idealised attributes and inspirational motivation but scoring lowest in individualised consideration. SE was rated positively overall (*M* = 3.70, SD = 0.73), with the opportunity subscale scoring highest (*M* = 4.09, SD = 0.83) and resources lowest (*M* = 3.36, SD = 0.94). Clinical leadership was perceived highly (*M* = 4.07, SD = 0.64), with MOW rated most favourably (*M* = 4.27, SD = 0.69). Self‐reported patient AEs occurred infrequently (*M* = 1.47, SD = 0.52), with medication errors and patient falls least reported (*M* = 1.27, SD = 0.58), while pressure ulcers (*M* = 1.61, SD = 0.71) and nosocomial infections (*M* = 1.59, SD = 0.73) were more common. QOC was rated favourably (*M* = 3.12, SD = 0.51), with nurses expressing moderate confidence in patients’ postdischarge self‐management (*M* = 3.07, SD = 0.76). These findings highlight strengths in leadership and care quality, alongside ongoing challenges with resource availability and AE management.

**TABLE 3:** Mean scores for survey instruments and their subscales: transformational leadership (MLQ), structural empowerment (CWEQ‐II), clinical leadership (CLS) and two measures of patient care (frequency of patient adverse events and quality of nursing care) (means and standard deviations).

	**Scale range (0–4)**	**Overall mean scores**

Main scale	Transformational leadership (0–4)	3.01 (0.82)
Subscales	Idealised attributes	3.04 (0.82)
	Idealised behaviour	3.01 (0.84)
	Inspirational motivation	3.04 (0.87)
	Intellectual stimulation	3.00 (0.89)
	Individualised consideration	2.94 (0.93)

	**Scale range (1–5)**	**Overall mean scores**

Main scale	Structural empowerment	3.70 (0.73)
Subscales	Information	3.65 (0.91)
	Support	3.71 (0.90)
	Resources	3.36 (0.94)
	Opportunity	4.09 (0.83)

	**Scale range (1–5)**	**Overall mean scores**

Main scale	Clinical leadership survey (CLS)	4.07 (0.64)
Subscales	Challenging the process (CPP)	3.86 (0.78)
	Inspiring a shared vision (ISV)	4.02 (0.75)
	Enabling others to act (ICT)	4.10 (0.74)
	Modelling the way (MOW)	4.27 (0.69)
	Encouraging the heart (HER)	4.12 (0.74)

	**Scale range (1–4)**	**Overall mean scores**

Main scale	Self‐reported frequency of patient adverse events	1.47 (0.52)
Subscales	Patient received wrong medication or dose	1.27 (0.58)
	Patients fall with injuries	1.27 (0.58)
	Pressure ulcers after admission	1.61 (0.71)
	Healthcare‐associated (nosocomial) infections	1.59 (0.73)
	Complaints from patients or their families	1.62 (0.74)

	**Scale range (1–4)**	**Overall mean scores**

Main scale	Quality of nursing care	3.12 (0.51)
Subscales	How would you describe the quality of nursing care delivered to patients on your unit?	3.35 (0.66)
	How would you describe the quality of nursing care delivered on your last shift?	3.42 (0.64)
	Overall, over the past year would you say the quality of patient care in your hospital has deteriorated, remained the same or improved?	3.49 (0.87)
	How confident are you that your patients are able to manage their care when discharged from the hospital?	3.07 (0.76)

### 4.1. Associations of Head Nurse Demographics and Unit Characteristics

The findings from the multilevel analysis revealed several significant associations between head nurse demographics, unit characteristics and various outcomes (see Supporting 4 for detailed results). Regarding TL perceptions, Saudi head nurses were associated with a 0.36‐point decrease in ratings compared to Jordanian head nurses (*β* = −0.36, SE = 0.17), and head nurses with 3–5 years of experience showed a 0.38‐point decrease compared to those with over 10 years of experience (*β* = −0.38, SE = 0.15). For clinical leadership perceptions, nurses under male head nurses reported a 0.13‐point decrease in ratings compared to those under female head nurses (*β* = −0.13, SE = 0.06). Specialty units also demonstrated significant associations, with nurses in the ER and ICU reporting decreases of 0.19 points (*β* = −0.19, SE = 0.07) and 0.22 points (*β* = −0.22, SE = 0.06), respectively, compared to the general ward. For SE, nurses in the ICU reported ratings 0.20 points lower compared to those in the general ward (*β* = −0.20, SE = 0.09). Regarding adverse patient events, nurses in smaller units (one to eight beds) reported a 0.26‐point lower frequency of self‐reported AEs compared to nurses in larger units (19–27 beds; *β* = −0.26, SE = 0.09), while those in medium‐sized units (9–18 beds) reported a 0.13‐point lower frequency (*β* = −0.13, SE = 0.06). For QOC, nurses in smaller units (one to eight beds) reported ratings 0.24 points higher compared to those in larger units (*β* = 0.24, SE = 0.10). In contrast, nurses in the ER and ICU reported ratings 0.20 points (*β* = −0.20, SE = 0.069) and 0.25 points (*β* = −0.25, SE = 0.065) lower, respectively, compared to the general ward.

In contrast, head nurse gender, nationality and years of experience did not significantly influence SE, adverse patient events or QOC. Similarly, specialty units and staff shortages did not impact adverse patient events, while nationality and years of experience demonstrated no significant effects on clinical leadership perceptions (see Supporting Information 4).

### 4.2. Testing the Study Model and Hypotheses Using MSEM

Prior to the SEM analysis, the assumptions of normality, multicollinearity, sample size, reliability and other key diagnostics were checked to ensure the robustness of the analysis. Normality was assessed using skewness (−0.98–1.67) and kurtosis (−0.39–3.38), which fell within acceptable ranges (±2 for skewness, ±7 for kurtosis [50], and was deemed suitable for SEM. Multicollinearity was evaluated using tolerance and VIF values, which indicated no significant multicollinearity issues. The sample size exceeded the threshold required for statistical significance, ensuring adequate power. Composite reliability (CR) and Cronbach’s alpha confirmed the internal consistency of the constructs, and outlier diagnostics, linearity and homoscedasticity were all assessed and found satisfactory. These findings are in line with the fundamental assumptions of MSEM. Following these checks, confirmatory factor analysis (CFA) was conducted to ensure that the measurement models for all constructs demonstrated acceptable fit. The CFA results indicated that the measurement models for TL, SE, registered nurses’ clinical leadership, patient AEs and QOC exhibited excellent good to excellent fit indices (Supporting Information 3), with significant factor loadings across all indicators. The discriminant validity of the model was evaluated using interconstruct correlations and the square root of the average variance extracted (AVE). Each construct showed stronger correlations with its own latent variable than with others, supporting discriminant validity. The square roots of AVEs (diagonal values) exceeded the corresponding interconstruct correlations (off‐diagonal values), confirming that the constructs were conceptually distinct (Supporting Information 2). Detailed results of the discriminant validity, CFA, including fit indices and factor loadings, are provided in the Supporting Information 1, 2 and 3 sections.

The reliability of the model was assessed using squared multiple correlation (SMC), Cronbach’s alpha, CR and AVE. Most SMC values exceeded 0.50, reflecting strong reliability, although a few items demonstrated lower values (e.g., 0.257), indicating weaker construct representation. Cronbach’s alpha values ranged from 0.768 to 0.981, surpassing the 0.70 threshold for internal consistency. Similarly, all constructs achieved a CR value above 0.70, confirming the model’s reliability. These results demonstrate that the constructs are robust and reliable measures (Supporting Information 1).

**FIGURE 1:**
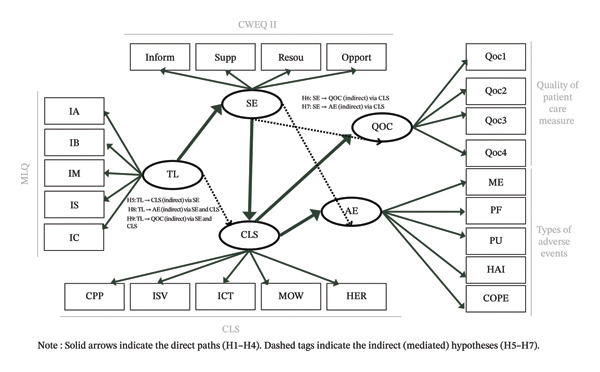
The hypothesised theoretical model.

Following validation of the measurement model, MSEM was employed to test the hypothesised relationships among the constructs. SEM allows for the simultaneous estimation of multiple regression equations, providing a comprehensive analysis of the direct and indirect relationships among the variables. The SEM model illustrated in Figure 1 explores the relationships between TL, SE, registered nurses’ clinical leadership and patient care outcomes, with nationality and gender included as control variables. The relationships between constructs are not limited to direct effects but also include the mediation role of some constructs. MSEM enables an examination of these relationships while accounting for the nested data structure, thus providing more accurate and reliable estimates. The fit of the structural model was assessed using similar fit indices as the multilevel CFA. This approach ensures that the model accurately represents the data at both the individual and group levels, providing robust support for the hypothesised relationships.

The findings of the structural equation modelling are presented in Table 4. The CFI and TLI scores were 0.955 and 0.948, respectively, indicating a good alignment between the model and the data. Additionally, the RMSEA value of 0.046 implied a good fit since it was below the ≤ 0.08 threshold. Furthermore, the SRMR value of 0.061 was well within acceptable limits, further supporting the adequacy of the model fit. While gender and nationality were significant in exploratory analyses across multiple variables, their effects became largely nonsignificant in the full SEM model after controlling for mediators (e.g., SE and clinical leadership), except for Indian nationality, which remained significant for clinical leadership (*p* = 0.033; see Table 5). This suggests that mediation diluted their direct impact, emphasising the mediators’ role in the relationships.

**TABLE 4:** Goodness‐of‐fit for multilevel SEM.

	CFI	TLI	RMSEA	SRMR
Recommended value	cutoff ≥ 0.90 0.95 + good fit	A value between 0.90 and 0.95 is considered marginal, above 0.95 is good	0.01, 0.05 and 0.08 to indicate excellent, good and adequate fit, respectively	0.08 or less good fit
SEM	0.955	0.948	0.046	0.061

**TABLE 5:** Path coefficients and significance for relationships among transformational leadership, structural empowerment, registered nurses’ clinical leadership (CLS), quality of care (QOC) and self‐reported frequency of patient adverse events in the SEM model.

Dependent variable	Independent variable	Hypothesis	Estimate	Std. err	*Z* value	CI (lower)	CI (upper)	*p* value	Standardised estimate	Hypothesis decision
SE	TL	H1	0.686	0.073	9.364	0.542	0.829	< 0.001	0.559	There is evidence of a statistically significant positive association between TL and SE
	Saudi		0.189	0.17	1.113	−0.144	0.523	0.266	−0.026	
	Filipino		−0.129	0.214	−0.602	−0.549	0.291	0.547	0.039	
	Indian		0.032	0.218	0.146	−0.395	0.459	0.884	0.006	
	Gender		0.305	0.183	1.666	−0.054	0.663	0.096	0.248	

CLS	SE	H2	0.502	0.058	8.673	0.389	0.616	< 0.001	0.52	There is evidence of a statistically significant positive association between SE and CLS
	Saudi		0.052	0.082	0.631	−0.109	0.212	0.528	0.005	
	Filipino		0.021	0.121	0.178	−0.215	0.258	0.859	0.011	
	Indian		0.283	0.133	2.136	0.023	0.543	0.033	0.06	
	Gender		0.101	0.101	1	−0.097	0.299	0.317	0.085	

QOC	CLS	H3	0.292	0.043	6.814	0.208	0.376	< 0.001	0.324	There is evidence of a statistically significant positive association between CLS and QOC
	Saudi		0.065	0.135	0.481	−0.2	0.33	0.631	0.051	
	Filipino		0.216	0.207	1.046	−0.189	0.621	0.296	0.015	
	Indian		0.023	0.235	0.099	−0.437	0.484	0.921	0.005	
	Gender		0.104	0.152	0.683	−0.194	0.402	0.495	0.098	

AE	CLS	H4	−0.128	0.034	−3.754	−0.194	−0.061	< 0.001	−0.149	There is evidence of a statistically significant negative association between CLS and AE
	Saudi		−0.062	0.255	−0.243	−0.561	0.437	0.808	−0.015	
	Filipino		−0.061	0.166	−0.369	−0.387	0.264	0.712	−0.015	
	Indian		0.268	0.315	0.851	−0.35	0.887	0.395	0.066	
	Gender		−0.091	0.237	−0.386	−0.556	0.373	0.7	−0.09	

*Note:* Transformational leadership (TL) was identified as the exogenous variable. Concurrently, structural empowerment (SE), the clinical leadership survey (CLS), self‐reported frequency of patient adverse events (AE) and quality of nursing care (QOC) were designated as endogenous variables.

Building on the results of the analysis, and while controlling for gender and nationality, the first four hypotheses (H1–H4) investigated direct effects within the model (Table 5).

The first hypothesis proposed that the TL of head nurses would be positively associated with an empowered healthcare system. This hypothesis found strong support, with TL being significantly positively associated with empowerment, reflected in an unstandardised estimate of 0.686 (SE = 0.073, *p* < 0.001) and a standardised estimate of *β* = 0.56. The moderate standardised estimate indicated a moderate association, suggesting that TL was meaningfully associated with SE. The second hypothesis suggested that SE would be positively associated with greater clinical leadership among registered nurses. This hypothesis was supported, with SE being significantly positively associated with registered nurses’ clinical leadership as evidenced by an unstandardised estimate of 0.502 (SE = 0.058, *p* < 0.001) and a standardised estimate of *β* = 0.52. This moderate standardised estimate points to a notable positive association. The third hypothesis posited that higher clinical leadership by registered nurses would be positively associated with improved patient care. This hypothesis was supported, as registered nurses’ clinical leadership was significantly positively associated with QOC, with an unstandardised estimate of 0.292 (SE = 0.043, *p* < 0.001) and a standardised estimate of *β* = 0.32. The low standardised estimate suggests a modest positive association. The fourth hypothesis proposed that higher clinical leadership by registered nurses would be negatively associated with the self‐reported frequency of patient AEs. This hypothesis was supported, as registered nurses’ clinical leadership was significantly negatively associated with patient AEs, with an unstandardised estimate of −0.128 (SE = 0.034, *p* < 0.001) and a standardised estimate of *β* = −0.15. The low standardised estimate indicates a mild negative association.

The subsequent hypotheses (H5–H9), summarised in Table 6, explored the mediating roles of SE and registered nurses’ clinical leadership in the relationships between TL and SE, registered nurses’ clinical leadership, QOC and patient AEs.

**TABLE 6:** Mediation of associations by structural empowerment and registered nurses’ clinical leadership (CLS) on the relationships between transformational leadership, quality of care (QOC) and adverse events (AEs).

Hypothesis	Independent variable	Mediator	Dependent variable	Estimate	Standard error	*z* value	*p* value	CI (lower)	CI (upper)	Standardised estimate	Hypothesis decision
H5	TL	SE	CLS	0.344	0.066	5.2	< 0.001	0.215	0.474	0.291	There is evidence that SE mediates the association between TL and CLS
H6	SE	CLS	QOC	0.147	0.029	5.071	< 0.001	0.09	0.203	0.169	There is statistically significant evidence that CLS mediates the association between SE and QOC
H7	SE	CLS	AE	−0.037	0.013	−2.967	0.003	−0.097	−0.031	−0.048	There is statistically significant evidence that CLS mediates the association between SE and AE
H8	TL	SE + CLS	AE	−0.044	0.013	−3.323	0.001	−0.07	−0.018	−0.043	There is statistically significant evidence that SE and CLS jointly mediate the association between TL and AE
H9	TL	SE + CLS	QOC	0.101	0.029	3.543	< 0.001	0.045	0.156	0.094	There is statistically significant evidence that SE and CLS jointly mediate the association between TL and QOC

The fifth hypothesis proposed that SE mediates the association between TL and the clinical leadership of registered nurses. This hypothesis found strong support, as TL was indirectly and positively associated with registered nurses’ clinical leadership through SE, with an unstandardised estimate of 0.344 (SE = 0.06, *p* < 0.001) and a standardised estimate of *β* = 0.291. The high standardised estimate indicates a strong mediating association of SE.

The sixth hypothesis suggested that clinical leadership by registered nurses mediates the association between SE and patient AEs. The data supported this hypothesis, showing that SE was indirectly and negatively associated with patient AEs via registered nurses’ clinical leadership, with an unstandardised estimate of −0.037 (SE = 0.013, *p* = 0.003) and a standardised estimate of *β* = −0.048. Although the standardised estimate was relatively low, indicating a weaker association, the mediation was statistically significant.

The seventh hypothesis posited that clinical leadership by registered nurses mediates the association between SE and the QOC. This hypothesis was supported by the data, as SE was indirectly and positively associated with QOC via registered nurses’ clinical leadership, with an unstandardised estimate of 0.147 (SE = 0.029, *p* < 0.001) and a standardised estimate of *β* = 0.169. This moderate standardised estimate suggests a moderate mediating association of registered nurses’ clinical leadership.

The eighth hypothesis proposed that SE and the clinical leadership of registered nurses together mediate the association between TL and patient AEs. The analysis supported this hypothesis, with TL being indirectly and negatively associated with patient AEs through SE and registered nurses’ clinical leadership, reflected in an unstandardised estimate of −0.044 (SE = 0.013, *p* = 0.001) and a standardised estimate of *β* = −0.043. While the standardised estimate is low, indicating a weaker association, the mediation is significant.

Finally, the ninth hypothesis suggested that SE and clinical leadership jointly mediate the association between TL and the QOC. This hypothesis is supported, as TL was indirectly and positively associated with QOC through SE and registered nurses’ clinical leadership, with an unstandardised estimate of 0.101 (SE = 0.029, *p* < 0.001) and a standardised estimate of *β* = 0.094. Although the standardised estimate is lower, indicating a weaker mediating association, the mediation remains significant.

These results highlight the crucial role of SE and registered nurses’ clinical leadership as mediators in the associations between the independent variables (TL and SE) and the dependent variables (registered nurses’ clinical leadership, QOC and patient AEs). While the strength of these mediating associations varies, the findings consistently underscore the importance of SE and registered nurses’ clinical leadership in shaping reported QOC and patient AE outcomes.

## 5. Discussion

This study examined how TL is associated with patient care outcomes, focussing on the mediating roles of SE and staff nurses’ clinical leadership. The findings reveal that SE and staff nurses’ clinical leadership jointly mediate the associations between TL and patient outcomes. TL was positively associated with staff nurses’ clinical leadership through SE, and staff nurses’ clinical leadership mediated the association between SE and both patient AEs and the QOC.

This study provides novel insights into how TL is associated with patient care outcomes through the mediating roles of SE and staff nurses’ clinical leadership, shifting the focus from simply confirming this association to understanding its underlying mechanisms. While previous research has primarily considered SE as a mediator between TL and quality of care outcomes [12, 51], this study uniquely establishes SE as a key mediator between TL and clinical leadership behaviours among nurses. Consistent with Kanter’s empowerment theory, which emphasises access to resources, information, support and opportunities for growth, the findings show that SE was associated with nurses’ assuming clinical leadership roles at the bedside, which in turn was linked with their ability to make decisions related to patient outcomes. This extends the work of Choi et al. and Asif et al. [3, 14], demonstrating that SE specifically mediates the relationship between TL and clinical leadership behaviours, and aligns with more recent systematic reviews highlighting empowerment as a crucial pathway through which TL improves both nurse and patient outcomes [10, 15].

In this study, clinical leadership played a pivotal role in mediating the relationship between SE and patient outcomes. Nurses who reported higher levels of SE also reported greater clinical leadership, which was associated with fewer AEs and better quality of care. This suggests that clinical leadership serves as a bridge between SE and patient outcomes, emphasising a sequential mediation from TL to SE to clinical leadership to patient outcomes. This extends the work of Boamah and Patrick et al. [16, 51] and is consistent with recent systematic reviews demonstrating that bedside clinical leadership enhances patient safety, strengthens team collaboration and contributes to improved care quality [11, 17]. As nurses engage in leadership behaviours fostered by an empowering work environment, they can better manage risks and prevent errors, contributing to higher care quality. The mediating role of clinical leadership highlights the importance of both SE and clinical leadership in improving patient care. Although some mediated associations were small in magnitude (e.g., standardised β < 0.15), such effects are common in multilevel clinical research where outcomes reflect many concurrent influences. Small, consistent associations can still be meaningful when embedded across units and sustained over time. For interpretability, one indirect path in this study (SE ⟶ clinical leadership ⟶ quality of care) corresponded to an unstandardised estimate of 0.147, indicating incremental but actionable change on the study scale.

Furthermore, although modest, the findings show that the combined mediating associations of SE and clinical leadership are key to understanding the broader links between TL and patient outcomes. While previous studies have examined these factors separately, this study highlights their joint mediating role in the associations of TL with quality of care and AEs. This dual mediation model suggests that TL may be associated with empowering nurses by providing both structural support and leadership opportunities, which were in turn linked to reported care delivery. This integrated view aligns with prior work emphasising the importance of empowered nurses in achieving positive patient outcomes [12, 29]. Practically, this implies that policies should prioritise scalable, routine practices that reinforce empowerment and bedside leadership (e.g., brief safety huddles, shared decision‐making, clear escalation protocols) and monitor incremental improvements using existing unit dashboards.

Nurses in this study rated their head nurses highly on TL and reported high levels of SE within their work environment, along with strong clinical leadership in their own roles. A significant positive relationship was found between TL and SE, emphasising the role of transformational leaders in fostering supportive, empowering work environments, a finding consistent with research by García‐Sierra and Fernández‐Castro, and Asif et al. [14, 52]. These leaders enhanced nurses’ access to resources, support and professional development, aligning with Kanter’s empowerment theory [13]. In turn, nurses who felt empowered through SE reported strong clinical leadership behaviours, highlighting that empowerment was associated with proactive leadership, effective communication and collaboration within healthcare teams. This link between SE and registered nurses’ clinical leadership suggests that when nurses have access to essential resources and support, they exhibit greater leadership at the bedside, which positively impacts patient outcomes by promoting quality care and reducing AEs [16, 53]. These findings underscore that TL, by fostering empowerment, is adaptable to diverse healthcare systems and cultural contexts globally. For instance, its principles have been shown to improve nurse performance and patient outcomes in countries with varying organisational structures and resource availability, such as the United Kingdom, the United States, China and Saudi Arabia [8, 54, 55].

The results of this study also revealed that demographic factors such as gender and nationality, as well as contextual factors like specialty units, were significantly associated with variations in nursing leadership and patient outcomes. Specifically, the multilevel exploratory analysis revealed that female head nurses demonstrated higher clinical leadership scores compared to male head nurses, suggesting that they may be more likely to be associated with stronger clinical leadership among their teams. This aligns with previous research indicating that female managers are often perceived as more supportive and interpersonally oriented, positively affecting employee performance [21, 56]. Regarding nationality, Jordanian and Indian head nurses exhibited higher levels of TL compared to their Saudi counterparts, indicating that cultural and professional experiences may impact leadership styles. Appelbaum et al. [57] similarly reported that a leader’s nationality can influence leadership practices due to cultural norms and expectations. Additionally, specialty units were associated with differences in perceptions of leadership and patient outcomes; nurses in General Wards and Operating Rooms reported higher levels of TL and SE, whereas Critical Care Units reported lower levels and higher incidences of AEs. However, it is important to note that Critical Care Units typically care for more severely ill patients, which may contribute to higher rates of AEs independently of leadership factors. Thus, while the study suggests a link between leadership and patient outcomes, the potential associations of patient acuity with these relationships warrant further investigation. This finding underscores the need for tailored leadership approaches in different clinical settings, as supported by Al‐Mugheed et al. [58] who found significant variations in patient safety attitudes across different units. These findings highlight the importance of considering demographic and unit‐specific factors when developing strategies to enhance nursing leadership and improve patient care outcomes.

However, the weaker association observed between clinical leadership and patient outcomes, compared to other relationships in the study, may reflect factors not directly captured by the clinical leadership measures. Challenges such as staffing ratios, skill mix and reliance on temporary staff are crucial determinants of patient outcomes [59]. Insufficient staffing or an inadequate skill mix can lead to increased workloads and burnout, affecting care quality and increasing the likelihood of AEs, regardless of clinical leadership levels [59]. Therefore, while clinical leadership is important, it operates within a broader systemic context where other factors, such as staffing and workload, are key determinants of patient outcomes.

In summary, the mediating roles of SE and clinical leadership offer a clear model for understanding how TL is associated with nurse behaviours and patient outcomes. By demonstrating that these factors together mediate the associations between TL and quality of care, this study contributes to the understanding of leadership dynamics in nursing. The combined associations of SE and clinical leadership appear to play an important role in reported care quality and fewer AEs.

### 5.1. Strengths and Limitations

This study is distinguished by its methodological robustness and comprehensive analytical approach. We utilised widely recognised and validated instruments, including the MLQ, the CWEQ‐II and the clinical leadership survey. These tools are globally recognised and have previously been used in similar population contexts. The study also benefited from a large, stratified sample size of 1038 registered nurses, ensuring an accurate representation of the general nursing population under study. The high response rate indicated strong participant engagement, further increasing the reliability and validity of the findings. Advanced statistical methods employed in this study included multilevel CFA and MSEM, implemented using IBM SPSS and R packages (lavaan, semPlot and semTools). These techniques allowed us to account for the hierarchical structure of the data (nurses nested within hospital units) and to simultaneously estimate multiple regression equations. This approach not only enhanced the accuracy of our estimates but also enabled us to uncover both direct and indirect relationships among TL, SE, clinical leadership and patient outcomes. In particular, the MSEM helped to elucidate the mediating mechanisms by which leadership is associated with outcomes, providing robust insights into the complex dynamics within the healthcare setting.

Additionally, the diverse nursing workforce in Saudi Arabia, comprising both Saudi and non‐Saudi nurses and head nurses from various nationalities, including Saudi, Indian, Filipino and Jordanian, enhances the external validity of the study. Despite being conducted within a single country’s health service, the diversity of participants suggests that the findings may be relevant to other healthcare systems with multicultural workforces. This diversity makes the insights globally relevant, and healthcare organisations worldwide can benefit from understanding and applying these mediating mechanisms to improve patient outcomes.

Limitations of the study include a reliance on self‐reported measures for SE, clinical leadership and quality of care, which may introduce bias, as responses could be influenced by personal experiences or specific unit dynamics rather than objective reality. Future research should consider using peer evaluations at the unit level to offer a more balanced perspective on clinical leadership and reduce the biases inherent in self‐assessment [60]. Another limitation of this study is the use of single‐source, cross‐sectional data, which may lead to CMB by potentially inflating relationships due to data collection from the same participants at a single point in time. Importantly, because the study employed a cross‐sectional design, the findings cannot establish causality. The observed relationships should be interpreted as correlational rather than causal, and future longitudinal or intervention‐based studies are needed to confirm causal pathways. Future studies could also mitigate CMB by using marker variables to identify shared variance or employing longitudinal designs to strengthen inference by tracking variables over time [49, 61, 62].

### 5.2. Implications for Nursing Practice, Administration, Policy and Education

This study highlights the importance of fostering TL among nurses to enhance organisational change and improve patient outcomes. Healthcare organisations should implement training programmes that combine theoretical and practical approaches to develop leadership skills at all levels. Leadership training is already a part of many nursing curricula; however, this should be supported by providing mentoring initiatives that prepare nurses for future leadership roles. Given that several effects were deemed modest but consistent, organisations should emphasise breadth and consistency of implementation rather than one‐off intensive initiatives and track incremental gains using existing quality indicators.

SE is pivotal for maximising nursing performance, engagement and patient care quality. Organisations must prioritise policies that provide nurses with essential resources, autonomy and professional support to enable them to lead effectively. Encouraging nurses to participate in committees and task forces will further build their leadership capacity and foster creativity in addressing organisational challenges.

This study has highlighted the importance of upholding gender inclusivity and equality within nursing leadership, aligning with initiatives like the NHS’s Equality, Diversity and Inclusion (EDI) Improvement Plan. This NHS plan emphasises an inclusive environment that allows all staff, particularly underrepresented groups, to thrive in leadership roles. Specifically, it advocates for policies that reduce barriers, promote diverse leadership and encourage equal participation across genders and backgrounds, recognising the positive impact on patient care and workforce cohesion [63].

Support for continuing education and professional development should be prioritised so that nurses aspiring to enhance their leadership practices can benefit from this. For instance, within the United Kingdom, the NHS programme ensures that nursing heads and leaders have access to the required training and supervision to positively impact their colleagues and peers in their future practice as nurse managers [64]. Similarly, the Institute of Medicine in the United States emphasises the importance of advanced nursing education and leadership training as part of its action‐oriented blueprint for the future of nursing, advocating for programmes that empower nurses to assume leadership roles in healthcare redesign [65]. The current study aligns with global recommendations to support nurses seeking leadership and growth opportunities, thereby enabling them to achieve their personal and professional development goals.

Addressing leadership gaps, particularly in specialty units, is vital for ensuring high‐quality patient care. Tailored interventions should focus on challenges unique to specific units, creating a supportive environment that motivates nurses to deliver superior care. Evidence‐based policies should account for factors like staff shortages and unit size to enhance the effectiveness of leadership strategies.

In summary, the key recommendations are as follows:

• Foster TL qualities to drive organisational change and improve patient outcomes.
• Implement leadership training programmes combining theory and practice.
• Further integrate leadership development into nursing education and mentoring programmes.
• Promote SE through autonomy, resources and professional support.
• Encourage nurses’ participation in committees and task forces for leadership development.
• Prioritise diversity and inclusivity in nursing leadership to improve decision‐making and innovation.
• Support continuing education and professional development to enhance leadership skills and career progression.
• Develop evidence‐based policies to support SE and address confounding factors.
• Focus on leadership development in specialty units to address unique challenges and improve patient outcomes.

### 5.3. Implications for Practice

TL is crucial for maximising nursing performance, engagement and patient care quality. Developing these leadership qualities can significantly enhance nurses’ ability to provide high‐quality care. To fully leverage these benefits, healthcare organisations should implement policies that promote SE by equipping nurses with essential resources, autonomy and professional support. Because several associations were deemed modest in this study, emphasis should be placed on embedding empowerment and bedside leadership behaviours into daily work, and monitoring steady, incremental improvement rather than expecting large, immediate changes.

Furthermore, supporting clinical leadership among nurses at the bedside appears vital. Greater involvement of nurses in clinical decision‐making was associated with more proactive roles in patient care, which in turn was linked to better reported patient outcomes and safety. Education and development programmes should prioritise TL training, incorporating practical and mentorship‐driven approaches to build leadership capabilities across all nursing levels. This focus will prepare nurses not only to lead effectively within their units but also to embrace TL as a core aspect of their practice.

## 6. Conclusion

TL is a crucial strategy in nursing, facilitating opportunities for growth and professional development. This research has revealed the specific mechanisms through which TL influences patient outcomes by highlighting the mediating roles of SE and clinical leadership. It has shown that TL not only provides nurses with SE, enhancing resource utilisation, information access and supportive environments but also fosters clinical leadership among head nurses and staff, leading to higher quality patient care and fewer AEs. Healthcare organisations can leverage these findings by developing evidence‐based leadership frameworks that integrate TL principles. Targeted initiatives should focus on training programmes that promote SE and bedside clinical leadership, addressing unit‐specific challenges such as staff shortages and varying leadership needs across specialties. Additionally, organisations should prioritise diversity in leadership by implementing policies that encourage inclusive participation across genders, cultural backgrounds and professional experiences. These strategies will help create an empowered and diverse nursing workforce capable of driving improvements in care delivery, fostering innovation and enhancing patient safety. By doing so, the full potential of TL can be realised, ensuring a more empowered and effective nursing workforce. This research provides a strong foundation for the creation of evidence‐based leadership frameworks, setting the stage for transformative changes in healthcare management and policy aimed at improving patient care outcomes.

## Author Contributions

Abdulaziz K. Alanazi contributed to the conception and design of the study, data collection, data analysis, interpretation of the findings and drafting of the manuscript. Susan A. Clarke, Marian Traynor and Clare McKeaveney contributed to the conception and design of the study, development of the research protocol and proposal, and provided supervision and critical revision throughout the research process and manuscript preparation. Lisa McFetridge contributed to review of the analysis, interpretation of the findings and critical revision of the manuscript.

## Funding

This research received no specific grant from any funding agency in the public, commercial or not‐for‐profit sectors. Abdulaziz K. Alanazi was supported by King Saud Medical City, Riyadh, Saudi Arabia, through sponsorship undertaken for his PhD studies.

## Disclosure

All scientific contents, interpretation and final wording were reviewed and approved by the authors, who take full responsibility for the manuscript. All authors read and approved the final manuscript.

## Conflicts of Interest

The main author is employed by King Saud Medical City (KSMC), the institution in which this research was conducted, and their doctoral study was sponsored by KSMC. This relationship was disclosed to the relevant ethics committees and nursing administration. To minimise any potential influence of this affiliation on the research process, several precautions were taken. First, data collection was anonymous, ensuring that participants’ responses were unidentifiable, thereby reducing any potential influence of the researcher’s position. Second, the survey was administered online, enabling participants to provide responses freely without any direct contact. Furthermore, data analyses and interpretations were performed objectively under the regular guidance of academic supervisors at Queen’s University Belfast (QUB) as well as experts in statistics. Model development and outputs were discussed and reviewed by the entire research team, ensuring that oversight was maintained throughout the process. These measures were undertaken to protect the integrity of the research and prevent bias.

## Supporting Information

Additional supporting information can be found online in the Supporting Information section.

## Supporting information


**Supporting Information** Supporting Data 1 details the validity and reliability of the measurement model using multilevel confirmatory factor analysis (CFA), including factor loadings, CR, AVE and Cronbach’s alpha for each construct. Supporting Data 2 presents the intercorrelation matrix used to assess discriminant validity across all study constructs in the full multilevel CFA model. Supporting Data 3 includes model fit indices (CFI, TLI, RMSEA, SRMR) for the confirmatory factor analysis of each latent construct in the study. Supporting Data 4 provides detailed multilevel regression models assessing the impact of head nurse demographics and unit characteristics on TL, SE, clinical leadership, patient AEs and quality of care.

## Data Availability

The data that support the findings of this study are available from the corresponding author upon reasonable request.
