# Supplementary material for: Linking Transformational Leadership to Patient Care Quality: The Role of Structural Empowerment and Registered Nurses’ Clinical Leadership
Source: J Nurs Manag. 2026 May 20;2026:7518764. doi: 10.1155/jonm/7518764 (PMC13191772; doi:10.1155/jonm/7518764)
Supplement: Supplementary file 1 — Supporting Information Supporting Data 1 details the validity and reliability of the measurement model using multilevel confirmatory factor analysis (CFA), including factor loadings, CR, AVE and Cronbach’s alpha for each construct. Supporting Data 2 presents the intercorrelation matrix used to assess discriminant validity across all study constructs in the full multilevel CFA model. Supporting Data 3 includes model fit indices (CFI, TLI, RMSEA, SRMR) for the confirmatory factor analysis of each latent construct in the study. Supporting Data 4 provides detailed multilevel regression models assessing the impact of head nurse demographics and unit characteristics on TL, SE, clinical leadership, patient AEs and quality of care. [file JONM-2026-7518764-s001.docx]

**Transformational Leadership and Patient Care Quality: Exploring the Mediating Roles of Structural Empowerment and Nurses’ Clinical Leadership – A Cross-Sectional Study**

**Supplementary Materials**

The following supplementary files are provided to support the findings of this study:

- Supplementary Data 1: Details the validity and reliability of the measurement model using multilevel confirmatory factor analysis (CFA), including factor loadings, CR, AVE, and Cronbach's alpha for each construct.
- Supplementary Data 2: Presents the inter-correlation matrix used to assess discriminant validity across all study constructs in the full multilevel CFA model.
- Supplementary Data 3: Includes model fit indices (CFI, TLI, RMSEA, SRMR) for the confirmatory factor analysis of each latent construct in the study.
- Supplementary Data 4: Provides detailed multilevel regression models assessing the impact of head nurse demographics and unit characteristics on transformational leadership, structural empowerment, clinical leadership, patient adverse events, and quality of care

# Supplementary Data 1: Validity and reliability for measured model using multilevel CFA

|  |  | **Factor**  **loading** | **z-value** | **p-value** | **R^2^** | **CR** | **Cronbach's**  **alpha** | **AVE** |
| --- | --- | --- | --- | --- | --- | --- | --- | --- |
| **TL** | IA | 0.937 | 18.89 | <.001 | 0.878 | 0.972 | 0.981 | 0.874 |
|  | IB | 0.904 | 18.28 | <.001 | 0.817 |  |  |  |
|  | IM | 0.948 | 20.01 | <.001 | 0.899 |  |  |  |
|  | IS | 0.953 | 19.82 | <.001 | 0.908 |  |  |  |
|  | IC | 0.931 | 21.07 | <.001 | 0.867 |  |  |  |
| **SE** | Information | 0.780 | 30.04 | <.001 | 0.608 | 0.840 | 0.823 | 0.558 |
|  | Support | 0.891 | 24.69 | <.001 | 0.794 |  |  |  |
|  | Resource | 0.702 | 18.98 | <.001 | 0.493 |  |  |  |
|  | Opportunity | 0.581 | 11.07 | <.001 | 0.338 |  |  |  |
| **CLS** | CPP | 0.737 | 17.51 | <.001 | 0.543 | 0.915 | 0.940 | 0.687 |
|  | ISV | 0.846 | 25.01 | <.001 | 0.716 |  |  |  |
|  | ICT | 0.898 | 28.22 | <.001 | 0.806 |  |  |  |
|  | MOW | 0.836 | 22.39 | <.001 | 0.699 |  |  |  |
|  | HER | 0.820 | 32.00 | <.001 | 0.672 |  |  |  |
| **AE** | ME | 0.729 | 11.75 | <.001 | 0.531 | 0.831 | 0.832 | 0.506 |
|  | PF | 0.746 | 12.00 | <.001 | 0.557 |  |  |  |
|  | PU | 0.692 | 17.09 | <.001 | 0.479 |  |  |  |
|  | HAI | 0.691 | 16.90 | <.001 | 0.477 |  |  |  |
|  | COPF | 0.698 | 13.18 | <.001 | 0.487 |  |  |  |
| **QOC** | Qoc1 | 0.871 | 22.83 | <.001 | 0.759 | 0.748 | 0.768 | 0.498 |
|  | Qoc2 | 0.815 | 15.94 | <.001 | 0.664 |  |  |  |
|  | Qoc3 | 0.507 | 8.92 | <.001 | 0.257 |  |  |  |
|  | Qoc4 | 0.558 | 12.36 | <.001 | 0.311 |  |  |  |
| *Transformational leadership (TL) was identified as the exogenous variable. Concurrently, structural empowerment (SE), the clinical leadership survey (CLS), the self-reported frequency of patient adverse events (AE), and quality of nursing care (QOC) were designated as endogenous variables.* | | | | | | | | |

# Supplementary Data 2: Inter-Correlation Matrix for Discriminant Validity of Full Model Using Multilevel CFA

|  | TL. | SE. | CLS. | AE. | QOC. |
| --- | --- | --- | --- | --- | --- |
| TL. | **0.935** | 0.536 | 0.348 | 0.008 | 0.228 |
| SE. | 0.536 | **0.747** | 0.508 | -0.102 | 0.412 |
| CLS | 0.348 | 0.508 | **0.829** | -0.146 | 0.318 |
| AE | 0.008 | -0.102 | -0.146 | **0.711** | -0.210 |
| QOC. | 0.228 | 0.412 | 0.318 | -0.210 | **0.706** |
| *The square root of average variance extracted (diagonal) of each construct and correlation with other constructs (off-diagonal).*  *Transformational leadership (TL) was identified as the exogenous variable. Concurrently, structural empowerment (SE), the clinical leadership survey (CLS), the self-reported frequency of patient adverse events (AE), and quality of nursing care (QOC) were designated as endogenous variables*. | | | | | |

# Supplementary Data 3: Measurement Model Fit Indices of CFA Models

| Construct | Items | CFI | TLI | RMSEA | SRMR |
| --- | --- | --- | --- | --- | --- |
|  | RECOMMENDED VALUE | cut-off ≥ 0.90 .95+ good fit | A value between .90 and .95 is considered marginal, above .95 is good | 0.01, 0.05, and 0.08 to indicate excellent, good, and adequate fit, respectively | .08 or less good fit |
| TL | IA,IB,IM,IS,IC | .992 | .983 | 0.069 | .007 |
| SE | Inform, Supp, Resources, Opport | .999 | .998 | .005 | .008 |
| CLS | CPP,ISV,ICT,MOW,HER | .956 | .913 | .144 | .023 |
| AE | ME,PF,PU,HAI,COPF | .964 | .928 | .081 | .030 |
| QOC | Qoc1,Qoc2,Qoc3,Qoc4 | .955 | .864 | .134 | .047 |

# Supplementary Data 4

**Effect of Level 2 Individual Demographic Factors on Transformational Leadership Outcomes**

|  | **Model 1 Null unit-level model** | | | **Model 2 HN Gender** | | | **Model 3 HN NATIONALITY** | | | **Model 4 Years of EXPERIENCE as HN** | | | **Model 5 speciality units** | | |
| --- | --- | --- | --- | --- | --- | --- | --- | --- | --- | --- | --- | --- | --- | --- | --- |
| **Fixed Effects** | **Coeff (95% CI)** | **S.E** | **P value** | **Coeff (95% CI)** | **S.E** | **P value** | **Coeff (95% CI)** | **S.E** | **P value** | **Coeff (95% CI)** | **S.E** | **P value** | **Coeff (95% CI)** | **S.E** | **P value** |
| **Intercept** | 3.01 [2.89, 3.12] | 0.06 | <.001 | 3.021 [2.87, 3.17] | 0.07 | <.001 | 3.20 [2.92, 3.48] | 0.14 | <.001 | 3.20 [2.95, 3.45] | 0.13 | <.001 | 3.10 [2.93, 3.26] | 0.08 | <.001 |
| **HN GENDER** |  |  |  |  |  |  |  |  |  |  |  |  |  |  |  |
| Male |  |  |  | -0.04 [-0.29, 0.20] | 0.13 | 0.74 |  |  |  |  |  |  |  |  |  |
| **HN NATIONALITY** |  |  |  |  |  |  |  |  |  |  |  |  |  |  |  |
| Saudi |  |  |  |  |  |  | -0.36 [-0.68, -0.03] | 0.17 | 0.03 |  |  |  |  |  |  |
| Filipino |  |  |  |  |  |  | -0.08 [-0.43, 0.27] | 0.18 | 0.653 |  |  |  |  |  |  |
| Indian |  |  |  |  |  |  | -0.06 [-0.48, 0.36] | 0.21 | 0.776 |  |  |  |  |  |  |
| **Years of EXPERIENCE as HN** |  |  |  |  |  |  |  |  |  |  |  |  |  |  |  |
| 1-2 years |  |  |  |  |  |  |  |  |  | -0.21 [-0.53, 0.12] | 0.17 | 0.22 |  |  |  |
| 3-5 years |  |  |  |  |  |  |  |  |  | -0.38 [-0.67, -0.10] | 0.15 | 0.009 |  |  |  |
| 6-10 years |  |  |  |  |  |  |  |  |  | 0.05 [-0.27, 0.36] | 0.16 | 0.78 |  |  |  |
| **speciality units** |  |  |  |  |  |  |  |  |  |  |  |  |  |  |  |
| ER |  |  |  |  |  |  |  |  |  |  |  |  | -0.23 [-0.52, 0.06] | 0.15 | 0.124 |
| ICU |  |  |  |  |  |  |  |  |  |  |  |  | -0.13 [-0.42, 0.16] | 0.15 | 0.379 |
| Cath lab |  |  |  |  |  |  |  |  |  |  |  |  | -0.52 [-1.19, 0.16] | 0.35 | 0.136 |
| OR |  |  |  |  |  |  |  |  |  |  |  |  | 0.06 [-0.65, 0.77] | 0.36 | 0.873 |
| haemodialysis |  |  |  |  |  |  |  |  |  |  |  |  | -0.05 [-0.48, 0.38] | 0.22 | 0.823 |
| **Variance Components** |  |  |  |  |  |  |  |  |  |  |  |  |  |  |  |
| Within Units Variance | 0.61 [0.55, 0.68] | 0.03 | <.001 | 0.61 [0.55, 0.68] | 0.03 | <.001 | 0.61 [0.55, 0.68] | 0.03 | <.001 | 0.61 [0.55, 0.69] | 0.03 | <.001 | 0.61 [0.55, 0.68] | 0.03 | <.001 |
| Between Units Variance | 0.07 [0.032, 0.15] | 0.03 | 0.01 | 0.07 [0.03, 0.15] | 0.03 | 0.01 | 0.05 [0.02, 0.11] | 0.02 | 0.016 | 0.03 [0.01, 0.09] | 0.02 | 0.066 | 0.06 [0.03, 0.12] | 0.02 | 0.012 |
| ICC |  |  |  |  |  |  |  |  |  |  |  |  |  |  |  |
| Between Units (units) variance | 0.10 10% |  |  | 0.10 10% |  |  | 0.07 7% |  |  | 0.05 5% |  |  | 0.08 08% |  |  |
| Within Units (nurses) variance | 0.90 90% |  |  | 0.90 90% |  |  | 0.93 93% |  |  | 0.95 95% |  |  | 0.92 92% |  |  |
| Log Likelihood | 1591.88 |  |  | 1591.77 |  |  | 1584.62 |  |  | 1580.595 |  |  | 1587.760 |  |  |
| AIC | 1597.88 |  |  | 1599.77 |  |  | 1596.62 |  |  | 1592.59 |  |  | 1603.76 |  |  |
| BIC | 1611.37 |  |  | 1617.76 |  |  | 1623.61 |  |  | 1619.58 |  |  | 1639.747 |  |  |

**Dependent variable:** transformational leadership. **HN:** Head Nurse. **Reference groups**: (HN Gender: Female); (HN Nationality: Jordanian); (YEARS OF EXPERIENCE AS HN: more than 10 years); (speciality units: GW)

**Effect of Level 2 Individual Demographic Factors on Structural Empowerment Outcomes**

|  | **Model 1 Null unit-level model** | | | **Model 2 HN Gender** | | | **Model 3 HN NATIONALITY** | | | **Model 4 Years of EXPERIENCE as HN** | | | **Model 5 speciality units** | | |
| --- | --- | --- | --- | --- | --- | --- | --- | --- | --- | --- | --- | --- | --- | --- | --- |
| **Fixed Effects** | **Coeff (95% CI)** | **S.E** | **P value** | **Coeff (95% CI)** | **S.E** | **P value** | **Coeff (95% CI)** | **S.E** | **P value** | **Coeff (95% CI)** | **S.E** | **P value** | **Coeff (95% CI)** | **S.E** | **P value** |
| **Intercept** | 3.71 [3.63, 3.78] | 0.04 | <.001 | 3.73 [3.64, 3.83] | 0.05 | <.001 | 3.69 [3.53, 3.85] | 0.08 | <.001 | 3.70 [3.57, 3.83] | 0.07 | <.001 | 3.79 [3.68, 3.90] | 0.06 | <.001 |
| **HN GENDER** |  |  |  |  |  |  |  |  |  |  |  |  |  |  |  |
| Male |  |  |  | -0.07 [-0.23, 0.09] | 0.08 | 0.364 |  |  |  |  |  |  |  |  |  |
| **HN NATIONALITY** |  |  |  |  |  |  | 0.01 [-0.06, 0.08] | 0.04 | 0.831 |  |  |  |  |  |  |
| **EXPERIENCE AS HN** |  |  |  |  |  |  |  |  |  | 0.00 [-0.02, 0.02] | 0.01 | 0.905 |  |  |  |
| **speciality units** |  |  |  |  |  |  |  |  |  |  |  |  |  |  |  |
| ER |  |  |  |  |  |  |  |  |  |  |  |  | -0.11 [-0.29, 0.07] | 0.09 | 0.215 |
| ICU |  |  |  |  |  |  |  |  |  |  |  |  | -0.20 [-0.37, -0.03] | 0.09 | 0.019 |
| Cath lab |  |  |  |  |  |  |  |  |  |  |  |  | -0.24 [-0.71, 0.23] | 0.24 | 0.313 |
| OR |  |  |  |  |  |  |  |  |  |  |  |  | -0.14 [-0.65, 0.37] | 0.26 | 0.592 |
| haemodialysis |  |  |  |  |  |  |  |  |  |  |  |  | 0.07 [-0.21, 0.34] | 0.14 | 0.621 |
| **Variance Components** |  |  |  |  |  |  |  |  |  |  |  |  |  |  |  |
| Within Units Variance | 0.51 [0.45, 0.56] | 0.03 | <.001 | 0.51 [0.45, 0.56] | 0.03 | <.001 | 0.51 [0.45, 0.56] | 0.03 | <.001 | 0.51 [0.45, 0.56] | 0.03 | <.001 | 0.51 [0.45, 0.56] | 0.03 | <.001 |
| Between Units Variance | 0.02 [0.01, 0.05] | 0.01 | 0.062 | 0.02 [0.01, 0.05] | 0.01 | 0.078 | 0.02 [0.01, 0.05] | 0.01 | 0.061 | 0.02 [0.006, 0.052] | 0.01 | 0.062 | 0.01 [0.002, 0.048] | 0.01 | 0.252 |
| ICC |  |  |  |  |  |  |  |  |  |  |  |  |  |  |  |
| Between Units (units) variance | 0.04 4% |  |  | 0.03 3% |  |  | 0.03 3% |  |  | 0.03 3% |  |  | 0.01 1% |  |  |
| Within Units (nurses) variance | 0.96 96% |  |  | 0.97 97% |  |  | 0.97 97% |  |  | 0.97 97% |  |  | 0.99 99% |  |  |
| Log Likelihood | 1446.81 |  |  | 1446.01 |  |  | 1446.76 |  |  | 1446.79 |  |  | 1440.72 |  |  |
| AIC | 1452.80 |  |  | 1454.01 |  |  | 1454.76 |  |  | 1454.79 |  |  | 1456.72 |  |  |
| BIC | 1466.30 |  |  | 1472.01 |  |  | 1472.75 |  |  | 1472.78 |  |  | 1492.71 |  |  |

**Dependent variable:** Structural empowerment. **HN:** Head Nurse; **Reference groups:** (speciality units: GW)

**Effect of Level 2 Individual Demographic Factors on Clinical Leadership Outcomes**

|  | **Model 1 Null unit-level model** | | | **Model 2 HN Gender** | | | **Model 3 HN NATIONALITY** | | | **Model 4 Years of EXPERIENCE as HN** | | | **Model 5 speciality units** | | |
| --- | --- | --- | --- | --- | --- | --- | --- | --- | --- | --- | --- | --- | --- | --- | --- |
| **Fixed Effects** | **Coeff (95% CI)** | **S.E** | **P value** | **Coeff (95% CI)** | **S.E** | **P value** | **Coeff (95% CI)** | **S.E** | **P value** | **Coeff (95% CI)** | **S.E** | **P value** | **Coeff (95% CI)** | **S.E** | **P value** |
| **Intercept** | 4.09 [4.02, 4.15] | 0.03 | <.001 | 4.13 [4.06, 4.21] | 0.04 | <.001 | 4.09 [3.97, 4.22] | 0.07 | <.001 | 4.12 [4.02, 4.23] | 0.05 | <.001 | 4.20 [4.12, 4.28] | 0.04 | <.001 |
| **HN GENDER** |  |  |  |  |  |  |  |  |  |  |  |  |  |  |  |
| Male |  |  |  | -0.13 [-0.24, -0.01] | 0.06 | 0.034 |  |  |  |  |  |  |  |  |  |
| **HN NATIONALITY** |  |  |  |  |  |  | -0.00 [-0.06, 0.05] | 0.03 | 0.891 |  |  |  |  |  |  |
| **EXPERIENCE AS HN** |  |  |  |  |  |  |  |  |  | -0.01 [-0.02, 0.01] | 0.01 | 0.41 |  |  |  |
| **speciality units** |  |  |  |  |  |  |  |  |  |  |  |  |  |  |  |
| ER |  |  |  |  |  |  |  |  |  |  |  |  | -0.19 [-0.32, -0.06] | 0.07 | 0.004 |
| ICU |  |  |  |  |  |  |  |  |  |  |  |  | -0.22 [-0.34, -0.11] | 0.06 | <.001 |
| Cath lab |  |  |  |  |  |  |  |  |  |  |  |  | -0.09 [-0.47, 0.30] | 0.2 | 0.66 |
| OR |  |  |  |  |  |  |  |  |  |  |  |  | -0.16 [-0.59, 0.26] | 0.22 | 0.445 |
| haemodialysis |  |  |  |  |  |  |  |  |  |  |  |  | -0.07 [-0.28, 0.14] | 0.11 | 0.538 |
| **Variance Components** |  |  |  |  |  |  |  |  |  |  |  |  |  |  |  |
| Within Units Variance | 0.40 [0.36, 0.45] | 0.02 | <.001 | 0.40 [0.36, 0.45] | 0.02 | <.001 | 0.40 [0.36, 0.45] | 0.02 | <.001 | 0.40 [0.36, 0.45] | 0.02 | <.001 | 0.40 [0.36, 0.44] | 0.02 | <.001 |
| Between Units Variance | 0.01 [0.00, 0.04] | 0.01 | 0.182 | 0.01 [0.00, 0.06] | 0.01 | 0.432 | 0.01 [0.01, 0.04] | 0.01 | 0.185 | 0.01 [0.002, 0.042] | 0.01 | 0.206 |  |  |  |
| ICC |  |  |  |  |  |  |  |  |  |  |  |  |  |  |  |
| Between Units (units) variance | 0.02 2% |  |  | 0.01 1% |  |  | 0.02 2% |  |  | 0.02 2% |  |  |  |  |  |
| Within Units (nurses) variance | 0.98 98% |  |  | 0.99 99% |  |  | 0.98 98% |  |  | 0.98 98% |  |  |  |  |  |
| Log Likelihood | 1285.78 |  |  | 1281.98 |  |  | 1285.76 |  |  | 1285.11 |  |  | 1273.499 |  |  |
| AIC | 1291.77 |  |  | 1289.97 |  |  | 1293.75 |  |  | 1293.05 |  |  | 1287.499 |  |  |
| BIC | 1305.27 |  |  | 1307.96 |  |  | 1311.75 |  |  | 1311.04 |  |  | 1318.987 |  |  |

**Dependent variable:** CLS. **HN**: Head Nurse. **Reference groups**: (HN Gender: Female); (speciality units: GW)

**Effect of Level 2 Individual Demographic Factors on Self-Reported Frequency of Patient Adverse Events Outcomes**

|  |  | **Model 1 Null unit-level model** | | | **Model 2 HN Gender** | | | **Model 3 HN NATIONALITY** | | | **Model 4 Years of EXPERIENCE as HN** | | | **MODEL 5 Number of Beds** | | |
| --- | --- | --- | --- | --- | --- | --- | --- | --- | --- | --- | --- | --- | --- | --- | --- | --- |
|  | **Fixed Effects** | **Coeff (95% CI)** | **S.E** | **P value** | **Coeff (95% CI)** | **S.E** | **P value** | **Coeff (95% CI)** | **S.E** | **P value** | **Coeff (95% CI)** | **S.E** | **P value** | **Coeff (95% CI)** | **S.E** | **P value** |
|  | **Intercept** | 1.47 [1.41, 1.53] | 0.03 | <.001 | 1.46 [1.39, 1.54] | 0.04 | <.001 | 1.41 [1.29, 1.53] | 0.06 | <.001 | 1.42 [1.26, 1.58] | 0.081 | <.001 | 1.57 [1.48, 1.65] | 0.04 | <.001 |
|  | **HN GENDER** |  |  |  |  |  |  |  |  |  |  |  |  |  |  |  |
|  | Male |  |  |  | 0.01 [-0.11, 0.14] | 0.06 | 0.826 |  |  |  |  |  |  |  |  |  |
|  | **HN NATIONALITY** |  |  |  |  |  |  | 0.03 [-0.03, 0.08] | 0.03 | 0.298 |  |  |  |  |  |  |
|  | **EXPERIENCE AS HN** |  |  |  |  |  |  |  |  |  | 0.02 [-0.04, 0.08] | 0.032 | 0.533 |  |  |  |
|  | **Number of Beds** |  |  |  |  |  |  |  |  |  |  |  |  |  |  |  |
|  | 1-8 |  |  |  |  |  |  |  |  |  |  |  |  | -0.26 [-0.43, -0.09] | 0.09 | 0.003 |
|  | 9-18 |  |  |  |  |  |  |  |  |  |  |  |  | -0.13 [-0.24, -0.02] | 0.06 | 0.021 |
|  | **speciality units** |  |  |  |  |  |  |  |  |  |  |  |  |  |  |  |
|  | **Staff shortages** |  |  |  |  |  |  |  |  |  |  |  |  |  |  |  |
|  | **Variance Components** |  |  |  |  |  |  |  |  |  |  |  |  |  |  |  |
|  | Within Units Variance | 0.26 [0.23, 0.29] | 0.02 | <.001 | 0.26 [0.23, 0.29] | 0.02 | <.001 | 0.26 [0.23, 0.29] | 0.02 | <.001 | 0.26 [0.23, 0.29] | 0.015 | <.001 | 0.26 [0.23, 0.29] | 0.01 | <.001 |
|  | Between Units Variance | 0.01 [0.01, 0.03] | 0.01 | 0.043 | 0.01 [0.00, 0.03] | 0.01 | 0.05 | 0.01 [0.01, 0.03] | 0.01 | 0.05 | 0.012 [0.005, 0.032] | 0.006 | 0.045 | 0.01 [0.002, 0.02] | 0.004 | 0.126 |
|  | ICC |  |  |  |  |  |  |  |  |  |  |  |  |  |  |  |
|  | Between Units (units) variance | 0.05 5% |  |  | 0.04 4% |  |  | 0.04 4% |  |  | 0.04 4% |  |  | 0.03 3% |  |  |
|  | Within Units (nurses) variance | 0.95 95% |  |  | 0.96 96% |  |  | 0.96 96% |  |  | 0.96 96% |  |  | 0.97 97% |  |  |
|  | Log Likelihood | 1010.50 |  |  | 1010.45 |  |  | 1009.44 |  |  | 1010.11 |  |  | 1001.54 |  |  |
|  | AIC | 1016.49 |  |  | 1018.45 |  |  | 1017.43 |  |  | 1018.11 |  |  | 1011.54 |  |  |
|  | BIC | 1029.99 |  |  | 1036.44 |  |  | 1035.42 |  |  | 1036.10 |  |  | 1034.03 |  |  |

**Dependent variable:** AE. **HN:** Head Nurse. **Reference groups**: (Number of beds: 19-27)

|  | **Model 6 speciality units** | | | **Model 7 Staff shortages** | | |
| --- | --- | --- | --- | --- | --- | --- |
| **Fixed Effects** | **Coeff (95% CI)** | **S.E** | **P value** | **Coeff (95% CI)** | **S.E** | **P value** |
| **Intercept** | 1.50 [1.41, 1.58] | 0.045 | <.001 | 1.41 [1.25, 1.56] | 0.077 | <.001 |
| **HN GENDER** |  |  |  |  |  |  |
| Male |  |  |  |  |  |  |
| **HN NATIONALITY** |  |  |  |  |  |  |
| **EXPERIENCE AS HN** |  |  |  |  |  |  |
| **Number of Beds** |  |  |  |  |  |  |
| 1-8 |  |  |  |  |  |  |
| 9-18 |  |  |  |  |  |  |
| **speciality units** | -0.02 [-0.06, 0.03] | 0.022 | 0.432 |  |  |  |
| **Staff shortages** |  |  |  | 0.02 [-0.03, 0.06] | 0.022 | 0.394 |
| **Variance Components** |  |  |  |  |  |  |
| Within Units Variance | 0.26 [0.23, 0.29] | 0.015 | <.001 | 0.26 [0.23, 0.29] | 0.015 | <.001 |
| Between Units Variance | 0.011 [0.004, 0.031] | 0.006 | 0.054 | 0.011 [0.004, 0.031] | 0.006 | 0.062 |
| ICC |  |  |  |  |  |  |
| Between Units (units) variance | 0.04 4% | 0.04 4% |  | 0.04 4% |  |  |
| Within Units (nurses) variance | 0.96 96% | 0.96 96% |  | 0.96 96% |  |  |
| Log Likelihood | 1009.90 |  |  | 1009.82 |  |  |
| AIC | 1017.90 |  |  | 1017.82 |  |  |
| BIC | 1035.90 |  |  | 1035.81 |  |  |

**Dependent variable:** AE. **HN:** Head Nurse. **Reference groups**: (Number of beds: 19-27)

**Effect of Level 2 Individual Demographic Factors on Quality of Patient Care Outcomes**

|  |  | **Model 1 Null unit-level model** | | | **Model 2 HN Gender** | | | **Model 3 HN NATIONALITY** | | | **Model 4 Years of EXPERIENCE as HN** | | | **MODEL 5 Number of Beds** | | |
| --- | --- | --- | --- | --- | --- | --- | --- | --- | --- | --- | --- | --- | --- | --- | --- | --- |
|  | **Fixed Effects** | **Coeff (95% CI)** | **S.E** | **P value** | **Coeff (95% CI)** | **S.E** | **P value** | **Coeff (95% CI)** | **S.E** | **P value** | **Coeff (95% CI)** | **S.E** | **P value** | **Coeff (95% CI)** | **S.E** | **P value** |
|  | **Intercept** | 3.37 [3.30, 3.43] | 0.03 | <.001 | 3.41 [3.33, 3.48] | 0.04 | <.001 | 3.43 [3.30, 3.56] | 0.07 | <.001 | 3.43 [3.32, 3.53] | 0.06 | <.001 | 3.32 [3.22, 3.42] | 0.05 | <.001 |
|  | **HN GENDER** |  |  |  |  |  |  |  |  |  |  |  |  |  |  |  |
|  | Male |  |  |  | -0.12 [-0.24, 0.01] | 0.07 | 0.073 |  |  |  |  |  |  |  |  |  |
|  | **HN NATIONALITY** |  |  |  |  |  |  | -0.03 [-0.09, 0.03] | 0.03 | 0.279 |  |  |  |  |  |  |
|  | **EXPERIENCE AS HN** |  |  |  |  |  |  |  |  |  | -0.01 [-0.02, 0.00] | 0.01 | 0.161 |  |  |  |
|  | **NUMBER OF BEDS** |  |  |  |  |  |  |  |  |  |  |  |  |  |  |  |
|  | 1-8 |  |  |  |  |  |  |  |  |  |  |  |  | 0.24 [0.04, 0.43] | 0.1 | 0.018 |
|  | 9-18 |  |  |  |  |  |  |  |  |  |  |  |  | 0.03 [-0.10, 0.16] | 0.07 | 0.665 |
|  | **speciality units** |  |  |  |  |  |  |  |  |  |  |  |  |  |  |  |
|  | ER |  |  |  |  |  |  |  |  |  |  |  |  |  |  |  |
|  | ICU |  |  |  |  |  |  |  |  |  |  |  |  |  |  |  |
|  | Cath lab |  |  |  |  |  |  |  |  |  |  |  |  |  |  |  |
|  | OR |  |  |  |  |  |  |  |  |  |  |  |  |  |  |  |
|  | haemodialysis |  |  |  |  |  |  |  |  |  |  |  |  |  |  |  |
|  | **Staff shortages** |  |  |  |  |  |  |  |  |  |  |  |  |  |  |  |
|  | **Variance Components** |  |  |  |  |  |  |  |  |  |  |  |  |  |  |  |
|  | Within Units Variance | 0.30 [0.27, 0.34] | 0.02 | <.001 | 0.30 [0.27, 0.34] | 0.02 | <.001 | 0.30 [0.27, 0.34] | 0.02 | <.001 | 0.30 [0.27, 0.34] | 0.02 | <.001 | 0.30 [0.27, 0.34] | 0.02 | <.001 |
|  | Between Units Variance | 0.02 [0.01, 0.04] | 0.01 | 0.033 | 0.01 [0.00, 0.04] | 0.01 | 0.076 | 0.02 [0.01, 0.04] | 0.01 | 0.039 | 0.01 [0.005, 0.038] | 0.01 | 0.045 | 0.01 [0.004, 0.033] | 0.006 | 0.068 |
|  | ICC |  |  |  |  |  |  |  |  |  |  |  |  |  |  |  |
|  | Between Units (units) variance | 0.05 5% |  |  | 0.04 4% |  |  | 0.05 5% |  |  | 0.05 5% |  |  | 0.04 4% |  |  |
|  | Within Units (nurses) variance | 0.95 95% |  |  | 0.96 96% |  |  | 0.95 95% |  |  | 0.95 95% |  |  | 0.96 96% |  |  |
|  | Log Likelihood | 1111.24 |  |  | 1108.42 |  |  | 1110.10 |  |  | 1109.37 |  |  | 1105.96 |  |  |
|  | AIC | 1117.23 |  |  | 1116.41 |  |  | 1118.09 |  |  | 1117.26 |  |  | 1115.96 |  |  |
|  | BIC | 1130.73 |  |  | 1134.40 |  |  | 1136.08 |  |  | 1135.25 |  |  | 1138.45 |  |  |

**Dependent variable:** QOC. **Reference groups**: (Number of beds: 19-27); (speciality units: GW)

|  |  | **Model 6 speciality units** | | | **Model 7 Staff shortages** | | |
| --- | --- | --- | --- | --- | --- | --- | --- |
|  | **Fixed Effects** | **Coeff (95% CI)** | **S.E** | **P value** | **Coeff (95% CI)** | **S.E** | **P value** |
|  | **Intercept** | 3.47 [3.39, 3.56] | 0.042 | <.001 | 3.37 [3.20, 3.55] | 0.089 | <.001 |
|  | **HN GENDER** |  |  |  |  |  |  |
|  | Male |  |  |  |  |  |  |
|  | **HN NATIONALITY** |  |  |  |  |  |  |
|  | **EXPERIENCE AS HN** |  |  |  |  |  |  |
|  | **NUMBER OF BEDS** |  |  |  |  |  |  |
|  | 1-8 |  |  |  |  |  |  |
|  | 9-18 |  |  |  |  |  |  |
|  | **speciality units** |  |  |  |  |  |  |
|  | ER | -0.20 [-0.34, -0.07] | 0.069 | 0.003 |  |  |  |
|  | ICU | -0.25 [-0.37, -0.12] | 0.065 | <.001 |  |  |  |
|  | Cath lab | -0.03 [-0.39, 0.33] | 0.184 | 0.87 |  |  |  |
|  | OR | -0.42 [-0.81, -0.03] | 0.2 | 0.037 |  |  |  |
|  | haemodialysis | -0.02 [-0.23, 0.19] | 0.106 | 0.865 |  |  |  |
|  | **Staff shortages** |  |  |  | -0.003 [-0.05, 0.05] | 0.025 | 0.918 |
|  | **Variance Components** |  |  |  |  |  |  |
|  | Within Units Variance | 0.30 [0.27, 0.34] | 0.017 | <.001 | 0.30 [0.27, 0.34] | 0.017 | <.001 |
|  | Between Units Variance | 0.005 [0.000, 0.055] | 0.006 | 0.432 | 0.016 [0.007, 0.041] | 0.008 | 0.033 |
|  | ICC |  |  |  |  |  |  |
|  | Between Units (units) variance | 0.01 1% |  |  | 0.05 5% |  |  |
|  | Within Units (nurses) variance | 0.99 99% |  |  | 0.95 95% |  |  |
|  | Log Likelihood | 1114.420 |  |  | 1111.23 |  |  |
|  | AIC | 1118.420 |  |  | 1119.23 |  |  |
|  | BIC | 1127.398 |  |  | 1137.22 |  |  |

**Dependent variable:** QOC. **Reference groups**: (Number of beds: 19-27); (speciality units: GW)
